# A review of biosensor for environmental monitoring: principle, application, and corresponding achievement of sustainable development goals

**DOI:** 10.1080/21655979.2022.2095089

**Published:** 2023-06-28

**Authors:** Chi-Wei Huang, Chitsan Lin, Minh Ky Nguyen, Adnan Hussain, Xuan-Thanh Bui, Huu Hao Ngo

**Affiliations:** a Department of Marine Environmental Engineering, National Kaohsiung University of Science and Technology, Kaohsiung, Taiwan; b Ph.D. Program in Maritime Science and Technology, College of Maritime, National Kaohsiung University of Science and TechnologyPh.D. Program in Maritime Science and Technology, Kaohsiung, Taiwan; c Ph. D. Program of Aquatic Science and Technology, College of Hydrosphere Science, National Kaohsiung University of Science and Technology, Kaohsiung, Taiwan; d Department Water Science & Technology, Key Laboratory of Advanced Waste Treatment Technology, Ho Chi Minh City University of Technology (HCMUT), Vietnam National University Ho Chi Minh (VNU-HCM), Ho Chi Minh City, Vietnam; e Department Water Science & Technology, Faculty of Environment & Natural Resources, Ho Chi Minh City University of Technology (HCMUT), Ho Chi Minh City, Vietnam; f Department Water Science & Technology, Centre for Technology in Water and Wastewater, School of Civil and Environmental Engineering, Faculty of Engineering and Information Technology, University of Technology Sydney, Sydney NSW, Australia

**Keywords:** Biosensor, bioavailability, sustainable development goals (SDGs), heavy metals, organic pollutants

## Abstract

Human health/socioeconomic development is closely correlated to environmental pollution, highlighting the need to monitor contaminants in the real environment with reliable devices such as biosensors. Recently, variety of biosensors gained high attention and employed as *in-situ* application, in real-time, and cost-effective analytical tools for healthy environment. For continuous environmental monitoring, it is necessary for portable, cost-effective, quick, and flexible biosensing devices. These benefits of the biosensor strategy are related to the Sustainable Development Goals (SDGs) established by the United Nations (UN), especially with reference to clean water and sources of energy. However, the relationship between SDGs and biosensor application for environmental monitoring is not well understood. In addition, some limitations and challenges might hinder the biosensor application on environmental monitoring. Herein, we reviewed the different types of biosensors, principle and applications, and their correlation with SDG 6, 12, 13, 14, and 15 as a reference for related authorities and administrators to consider. In this review, biosensors for different pollutants such as heavy metals and organics were documented. The present study highlights the application of biosensor for achieving SDGs. Current advantages and future research aspects are summarized in this paper.

**Abbreviations:** ATP: Adenosine triphosphate; BOD: Biological oxygen demand; COD: Chemical oxygen demand; Cu-TCPP: Cu-porphyrin; DNA: Deoxyribonucleic acid; EDCs: Endocrine disrupting chemicals; EPA: U.S. Environmental Protection Agency; Fc-HPNs: Ferrocene (Fc)-based hollow polymeric nanospheres; Fe_3_O_4_@3D-GO: Fe_3_O_4_@three-dimensional graphene oxide; GC: Gas chromatography; GCE: Glassy carbon electrode; GFP: Green fluorescent protein; GHGs: Greenhouse gases; HPLC: High performance liquid chromatography; ICP-MS: Inductively coupled plasma mass spectrometry; ITO: Indium tin oxide; LAS: Linear alkylbenzene sulfonate; *LIG*: Laser-induced graphene; LOD: Limit of detection; ME: Magnetoelastic; MFC: Microbial fuel cell; MIP: Molecular imprinting polymers; MWCNT: Multi-walled carbon nanotube; MXC: Microbial electrochemical cell-based; NA: Nucleic acid; OBP: Odorant binding protein; OPs: Organophosphorus; PAHs: Polycyclic aromatic hydrocarbons; PBBs: Polybrominated biphenyls; PBDEs: Polybrominated diphenyl ethers; PCBs: Polychlorinated biphenyls; PGE: Polycrystalline gold electrode; photoMFC: photosynthetic MFC; POPs: Persistent organic pollutants; rGO: Reduced graphene oxide; *RNA*: Ribonucleic acid; SDGs: Sustainable Development Goals; SERS: Surface enhancement Raman spectrum; SPGE: Screen-printed gold electrode; SPR: Surface plasmon resonance; SWCNTs: single-walled carbon nanotubes; TCPP: Tetrakis (4-carboxyphenyl) porphyrin; TIRF: Total internal reflection fluorescence; TIRF: Total internal reflection fluorescence; TOL: Toluene-catabolic; TPHs: Total petroleum hydrocarbons; UN: United Nations; VOCs: Volatile organic compounds

## Graphical abstract

**Figure:**
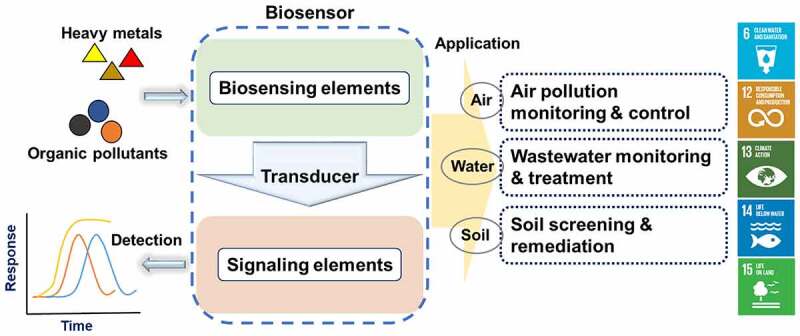

## Highlights

- Biosensors are robust in both specific and total responses to environmental pollutants
- Promising prospects for achieving on-site monitoring and real-time environmental data
- Biosensors illustrate a reliable, simple, effective, and fast method for monitoring pollution
- Biosensors consume less energy and leave a smaller carbon footprint
- High performing biosensors make a solid contribution to SDGs

## 1. Introduction

Detection and monitoring of pollutants in the environment is crucial for assessing the harmful effects of potential toxicants to people, flora, and fauna [1–3]. Chemical analysis using instruments such as high performance liquid chromatography (HPLC), gas chromatography (GC), and inductively coupled plasma-mass spectrometry (ICP-MS) has been traditionally used for monitoring environmental water or soil samples for their sensitivity and accuracy [4]. To further understand the bioavailability of environmental pollutants, biosensors using biological sensor to detect such pollutants have been invented, and they have features of low-cost, energy-saving, and feasibility for real-time *in situ* monitoring [4,5]. Different types of biosensors were successfully applied for specific or nonspecific detection of heavy metals [6,7] and organic pollutants [1,8]. In groundwater and river water samples, the bioavailable and toxic fractions of metals and organic pollutants such as Cd and toluene could be successfully evaluated using biosensors [9,10]. Moreover, bioavailability of the pollutants such as Hg and phenanthrene was revealed by the use of biosensors [11,12], suggesting the useful application of biosensors on bioavailability assessment of pollutants in the environmental samples.

Biosensor is an analytical strategy for pollutants based on biotechnology, and consists of elements for signal transducer producing detectable or quantifiable signals when sensing pollutants [6,13]. Types of biosensor include cell-free-based and whole-cell-based, nonspecific and specific ones according to the biological elements and sensing ability [14]. The bioavailability and toxic effects can be established by whole-cell-based biosensor compared with a cell-free one [14,15]. For example, microorganisms possess various responsive mechanisms to cope with environmental stress, organic pollutants, and heavy metals, which are associated with different regulatory genes and proteins. The Cd detecting protein cadC regulator could be used in biosensor for Cd detection [16]. In addition, the benzene metabolizing regulatory protein would be useful in recognizing benzene-like organic pollutants [17]. Those regulatory systems interacting with environmental pollutants are the key factors for distinguishing specific pollutants from others [16,17]. However, pollutants with similar structure might hamper the selectivity of biosensors, such as the benzene regulatory protein targeting toluene, ethylbenzene, toluene, and xylene [17]. Therefore, the choice of regulatory systems or recognizing elements would greatly affect the selectivity of specific biosensors. Currently, biosensors targeting heavy metals, pesticides, or pharmaceuticals show the advantages of biosensor such as portability, ease of use, and saving time [18,19]. Recent studies have highlighted the advances made in sensitivity, stability, selectivity, and their contribution to environmental monitoring [1,8].

It has been shown that in clinical laboratories, the consumption of electricity, water, and gas as well as the waste produced are the key factors generating large carbon footprints [20]. Energy use was also the major contributor to greenhouse gases (GHGs) emissions [21,22]. It is suggested that potential carbon reduction could be achieved by partially using biosensors in pollutants analysis due to their low energy requirements. One review has suggested that the contribution of using biosensor for human health on SDGs [23]. However, discussions about the application of biosensor on environmental monitoring and its relationship between sustainable development goals (SDGs) are scarce. Biosensors technology is anticipated to be powerful tool for monitoring the environmental pollutants for its advantages on sustainable development. In this article, different types of biosensors, principles of biosensor construction as well as the application and performance are reviewed. The relationship between biosensors and SDGs is also noted. This review will also help to achieve the SDGs since biosensors play a significant role to achieve the SDG 6, 12, 13, 14, and 15.

## 2. Types of biosensors

### 2.1 Cell-free biosensors

**Figure 1.**
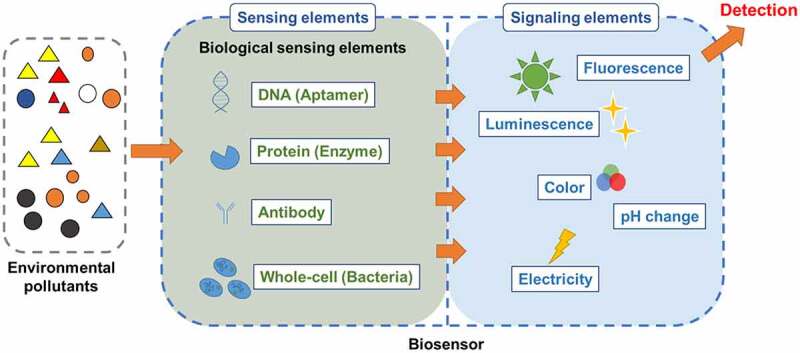
General mechanisms of biosensor for pollutants detection. The basic construction of biosensor contains sensing elements and signaling elements. The biological-sensing elements including DNA (aptamer), protein (enzyme), antibody, and whole-cell (bacteria) are able to recognize the environmental pollutants such as heavy metals and organic pollutants. The signaling elements would be triggered by sensing elements and produce the different signals such as fluorescence, luminescence, color, pH change, or electricity that could be measured or detected by the operators.

**Figure 2.**
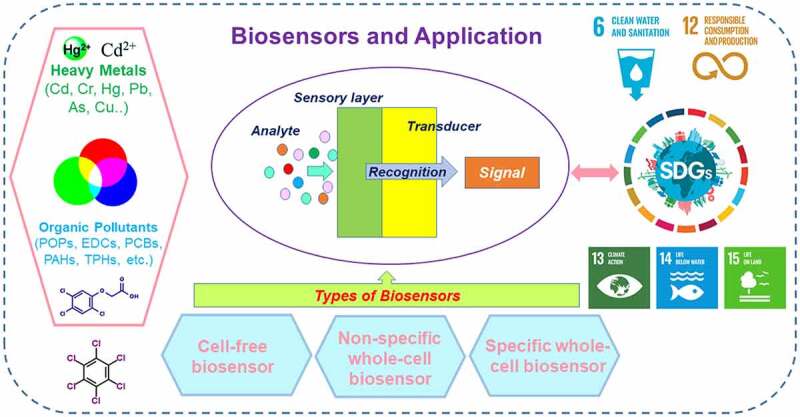
The types of biosensors and the applications on SDGs achievements. The different environmental pollutants including heavy metals and organic pollutants could trigger the sensing, transducing, and signaling of biosensors. The different types of biosensors include cell-free biosensor, nonspecific whole-cell biosensor, and specific whole-cell biosensor, which is categorized mainly by their sensing elements and selectivity. Those biosensors detecting the environmental pollutants could help achieving SDG 6, 12, 13, 14, 15.

The general mechanism of biosensors for environmental monitoring is displayed in Figure 1. The different types of biosensors and the application on SDGs achievement were shown in Figure 2. The sensing elements of cell-free biosensors include deoxyribonucleic acid (DNA), protein, and aptamer. DNA-based biosensors could be used for monitoring heavy metals such as arsenic and lead made possible by changes in the specific DNA structure and oxidative damage [14,24]. Sulfur on the protein structure reacts easily with toxicants like arsenic and the resulting inhibition of enzyme activities can help detect toxic metals like arsenic [25,26]. Other enzyme such as acetylcholinesterase could also be used in inhibition-based biosensor, which is contributed from the binding of organic P pesticides with acetylcholinesterase inhibiting the further signal producing from the reaction of acetylcholinesterase [27]. However, the response of DNA and protein would involve other toxic substances and might lack selectivity to specific pollutants. To enhance the selectivity of target chemicals, aptamer, an artificial single-strand DNA or ribonucleic acid (RNA), was developed for specific pollutants detection based on selective bonding [28]. Currently, aptamer-based biosensors coupled with nanomaterials have been employed in several studies for detecting heavy metals and organic pollutants such as pesticides [29].

### 2.2 Nonspecific whole-cell biosensors

Nonspecific whole-cell biosensors are constructed mainly by stress responsive genetic regulation, such as heat shock and DNA damage responses [6]. Information about bioavailability and toxicity can be revealed by nonspecific cell-based biosensors. The damage done to protein will trigger heat shock stress-related genes, which could be used as a sensing element of nonspecific cell-based biosensors for pollutants that damage protein [30–32]. The expression of SOS response genes enhanced by DNA damage could be incorporated in nonspecific sensing elements [33–36]. Indigenous bacteria and their activity as far as environmental stress is concerned could also serve as a nonspecific whole-cell biosensor. For example, sulfur oxidizing and iron oxidizing abilities in certain types of bacteria would be inhibited by toxic pollutants and could be used for acid samples [37–39]. As well, indigenous bacterium isolated from soil environments was successfully engineered with green fluorescent protein (GFP) signaling gene, which could help assess bioavailable heavy metals in the soils by the GFP signals [40]. The nonspecific biosensor based on the stress response provides an early warning of hazard presence in samples that could be harmful for microorganisms despite the lack of selectivity.

### 2.3 Specific whole-cell biosensors

**Figure 3.**
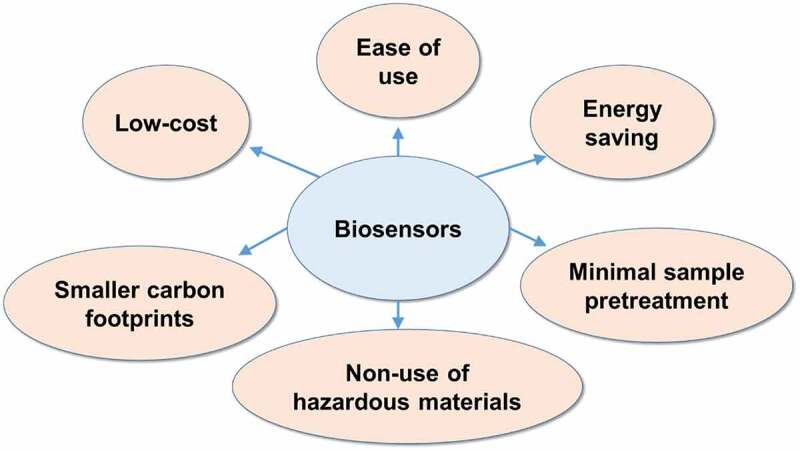
The advantages of biosensors on SDGs achievements. Several advantages of biosensors include low-cost, ease of use, saving energy, smaller carbon footprints, nonuse of hazardous materials, and minimal sample pretreatment compared with traditional physicochemical methods. These advantages are helpful to achieving SDGs such as SDG 6, 12, 13, 14, and 15, which relate to clean water, responsible consumption and production, climate action, and marine and terrestrial life.

Specific whole-cell biosensors are mostly based on the metabolism or detoxification genes in microorganisms [8,41]. For organic pollutants, toluene and xylene can be metabolized by toluene-catabolic (TOL) plasmid in bacteria harboring those regulatory genes [42]. The *xylR* and *xylS* genes on the TOL plasmid can serve in biosensors specifically designed for benzene-related compounds [43–45]. Other operons such as *nah, alkBAC*, and *DntR* were applied in a specific cell-based biosensor for naphthalene, linear alkanes, and pesticides [46–49]. For heavy metals, the sensing elements of specific cell-based biosensor belong to resistance genes instead of metabolized genes, which are located in the plasmid in microorganisms that could survive in toxic metal-rich environments [50]. Expression of the resistance genes including redox transformation, active transport, and intracellular/extracellular precipitation is regulated by intracellular metal level [51]. Both toxic metals and excessive essential metals could trigger the response of resistance or detoxification genes [52,53]. The *arsR* or *arsD* in *ars* operon for arsenic [14], the *cadC/A* in *cad* operon for cadmium [54], the *merR* in *mer* operon for mercury [6], and the *chrB* in *chr* operon for chromium were used in specific cell-based biosensors [55]. Cell-based biosensors for cadmium and mercury were able to detect other metal ions, such as copper, zinc, or lead, which reduces their selectivity [6,54]. *zntA* and *copA* promoter/operator evaluated the bioavailability of Zn and Cu [56]. *zntA* accounts for the removal of intracellular Zn and responds to Cd and Pb [57,58]. *copA* is responsible for the concentration of Cu and Ag in cells [59]. Regulatory proteins of *zntA* and *copA* belong to the MerR homologue, thereby making them responsible for Hg [60,61]. The *cnrYXH* gene was used to detect the bioavailability of Co and Ni in soil [62]. In addition, the Pb(II)-binding regulator PbrR in MerR family has the ability to recognize lead after being engineered in whole-cell biosensor [63]. The characteristics of biosensors of different types are summarized in Table 1. In addition, the advantages of biosensors on SDGs achievement are displayed in Figure 3.

**Table 1.** Characteristics of various type biosensors.

Biosensors	Selectivity	Response time	Sensitivity	Bioavailability
Cell-free biosensor	High	Fast	High	No
Nonspecific whole-cell biosensor	Low	Medium	Medium	Yes
Specific whole-cell biosensor	High	Medium	Medium	Yes

## 3. Principle of whole-cell biosensor construction

### 3.1 Toxicity-based nonspecific biosensor

The mechanisms of nonspecific biosensors based on toxicity include Microtox and adenosine triphosphate (ATP)-bioluminescence system, bacterial functions such as iron and sulfur oxidizing, and DNA damage or heat shock protein-related genes. The Microtox test has been devised using the bacterium *Vibrio fischeri* whose bioluminescence would diminish due to the toxicity and disrupted metabolism [64,65]. The mechanisms of bioluminescence-based inhibitory biosensors might be associated with the ATP reduction resulted from stress responsive mechanisms or cell death resulted from excessive environmental stress, which further decreased the bioluminescence by lower ATP and cell viability [17,66]. It is worth noting that the genes of bioluminescence can be genetically engineered in other lab-cultivable bacteria such as *E. coli*. Similarly, the constitutively expressed bioluminescence of these toxicity-based nonspecific biosensors would diminish upon the exposure of toxicants, thereby making them capable of quantifying the general toxicity equivalent of multiple pollutants [66]. As well, the ATP level reflected by bioluminescence could serve as an indicator of toxicity and metabolic disruption.

The modified *Saccharomyces cerevisiae* with substrate luciferin could serve as a reporter system based on ATP-bioluminescence for detecting nonspecific toxicity [67]. The metabolic cost might increase for stress response when the bacterial functions are inhibited by environmental pollutants, thereby reducing the amount of products of iron and sulfur oxidizing [37–39]. For example, the pH decrease and conductivity increase resulted from sulfur oxidation was inhibited by the toxicants [37], which might be due to the bacterial death or disrupted microbial functions. Based on the inhibition level, toxicity could be quantified by using iron and sulfur oxidizing bacteria [37–39]. The *recA* and its promoter responsible for DNA repair and maintenance were widely used in DNA damage-sensing biosensors for their response to genotoxic compounds and pharmaceuticals [34,68,69]. Recent studies have applied both *recA*-based and metabolic inhibition-based bioluminescence bacterial biosensor for pollutants such as toxic metals in urban rivers and seawater [70,71]. Similarly, the stress-inducible heat shock genes like *hsp70* and its promoter were used in biosensors for detecting nonspecific toxicity since the heat shock protein systems will be triggered not only by heat shock but also other environmental stressors, and then induce the downstream fused signals [72,73].

### 3.2 Metabolic inhibition-based bioluminescence bacterial biosensors

The metabolic inhibition-based bioluminescence bacterial biosensors have been developed and utilized in several pollutants detection [74]. Different signaling elements producing bioluminescence such as luciferase were used in the construction of bacterial biosensor, or naturally presented in bacteria that could be directly used in sensing [74]. The luciferase genes from bacteria and firefly have been suggested to be highly sensitive and broadly used as a reporter gene. The *luxCDABE* from bacterial operon is conservative in many bacteria species [75]. The *luxAB* is enough for producing the luminescence, while using the whole *luxCDABE* operon could reduce the amount of additional substrates that is needed for luminescence reaction [15]. The protein from *luxAB* is responsible for catalyzing long-chain aldehydes shown in the reaction below:

FMNH_2_ + R – CHO + O_2_ → FMN + H_2_O + RCOOH + *hν* (490 nm)

The *lucFF* gene from the firefly requires luciferin, O_2_, and ATP for bioluminescence [76]:

Firefly luciferin + O_2_ + ATP → oxyluciferin + AMP + P_i_ + *hν* (550–575 nm)

The bioluminescence would diminish in the presence of environmental pollutants due to the inhibited bioluminescence reaction. The ATP-dependent bioluminescence could be used in developing the metabolic inhibition-based biosensors for it can reflect the ATP decrease caused by environmental toxicants [67,74]. On the other hand, the energy-consuming *lux* operon reaction in *Vibro* spp. could also reflect the metabolic state of the microorganisms [66]. Therefore, the bioluminescence biosensors with *lux* operon such as Microtox (*Vibrio fischeri*) were applied in assessing the toxicity of environmental pollutants in river and sediment samples [71,77,78]. In addition to *Vibro* spp., the recombinant *Escherichia coli* harboring the *lux* operon could successfully detect the environmental pollutants, suggesting the feasibility of *lux* operon engineered in other bacterial species that could be easily cultured [8,79].

### 3.3 Microbial fuel cell-based biosensors

Microbial fuel cell (MFC)-based biosensors with single- or dual-chamber design were developed based on the oxidation of organic compounds and the corresponding electrical currents [80,81]. The dual chambers of MFC-based biosensors consist of an anaerobic anodic chamber where the oxidation happens and aerobic cathodic chamber, transferring electrons through circuits and protons through the ion-exchange membrane from anode to cathode [82]. MFC-based biosensors can detect the water quality and wastewater parameters such as biological oxygen demand (BOD) and toxins [81]. Compared with nonspecific whole-cell biosensors, MFC-based biosensors save more energy and are portable; they produce the electrical current and do not need signal transducers [82]. It has been reported that heavy metals such as copper and arsenic in wastewater were detected by MFC-based biosensor based on the toxicity associated inhibition [83]. The bacteria strains for organics degradation could be mixed culture from activated sludge, and the toxicants in wastewater could be sensed by the inhibited degradation [82]. In addition to pollutant-degrading bacteria, the electroactive bacteria such as *Geobacter* and *Shewanella* were shown to facilitate the current production in MFC-based biosensor [84]. Other bacterial strains in MFC-based biosensors for sensing specific pollutants were also reported in recent studies. For example, the linear alkylbenzene sulfonate could be simultaneously quantified and degraded dominantly by *Pseudomonas* species, generating electrical power using a MFC-based biosensor [85]. To increase the selectivity for specific detection, Cr(VI)-reducing bacterium *Exiguobacterium aestuarii* YC211 was cultured in a MFC-based biosensor system [86]. It can detect *in situ* Cr(VI) in industrial wastewater via the electrical currents generated from the reduction of Cr(VI) to Cr(III) [86], which suggests the possibility of specific detection and indigenous bacterium application in MFC-based biosensors. In addition, the genetically engineered *E. coli* was developed in MFC-based biosensors for detecting Zn(II) and Cu(II), which is accomplished by the recombining metal-sensing promoter, and the synthesis genes of riboflavin and porin [87,88]. The riboflavin and porin are responsible for facilitating electron transfer and improving cell membrane permeability, respectively [87,88].

### 3.4 Genetic modification using detoxification and metabolism genes

To detect specific pollutants, genetic methods using detoxification and metabolism genes to differentiate environmental pollutants have been developed. For organic compounds, the metabolic and utilization mechanisms and their regulatory genes were mainly used [8]. Recent research has combined genes and hosts from different bacterial species and used AtzR regulatory protein to detect cyanuric acid [89]. Biodegradable organic pollutants in the whole-cell biosensors trigger metabolic mechanisms and the downstream utilizing proteins or signals such as bioluminescence [8,90]. Subsequently, it is difficult to detect biodegradable organic pollutants since the metabolism mechanisms are unclear in the microorganisms. However, recent research has developed a whole-cell biosensor to detect persistent polybrominated diphenyl ethers (PBDEs) using genome-wide screening. It can find regulatory networks in the microorganisms [91], shedding light on the application of biosensors on organic pollutants that can only be degraded with difficulty.

For heavy metals, the resistance and detoxification mechanisms were applied in the sensing elements of specific whole-cell biosensors [92]. The regulatory systems consisted of regulatory protein that could bind metals and promoter regions that trigger downstream detoxification or signaling responses [92,93]. For example, *arsR* and the promoter region from *Geobacter sulfurreducens* could be genetically combined with a fluorescence reporter, usable for arsenic screening with the detection limit down to the ppb level [41]. Basically, the detoxification and metabolism operons were activated when the toxicants permeated in the cells, but it has been suggested that the two-component regulatory systems could be incorporated in bacterial biosensors for detecting extracellular pollutants [94]. The regulatory membrane protein has the ability to bind pollutants and then trigger downstream phosphorylation of the other regulator proteins that activate the signal transduction [94].

## 4. Application of biosensor to environmental monitoring

### 4.1 Heavy metals monitoring

Heavy metals pollution is usually discharged as a result of anthropogenic and industrial activities (e.g., refineries, metal works, mining, cement factories, smelting plants, etc.), and their pollution endangers human health and the environment [95–97]. Elements such as Fe, Cu, Mn, and Zn, then highly toxic Pb, Cr, Cd, As, Hg, etc, are very resistant to biodegradation. Heavy metals are transported into the environment, especially water sources and easily absorbed by living organisms. Due to their pronounced toxicity, these heavy metals are important for monitoring the environment (water, wastewater, air, solid waste, and organisms) [95,97,98]. Monitoring water contamination is essential for environmental conservation and the prevention of illnesses. Due to the accumulation in the environment (e.g., water, soil, sediment) and wildlife such as plants, animals over a long period of time, heavy metals pose a serious danger. Several methods have been devised to detect their concentrations and presence in the environmental matrix. Especially, biosensors can detect the levels of heavy metals and determine how much pollution they cause. Biosensors can easily detect the presence of heavy metals in order to regulate and manage water safety and quality.

The DNA probe is used as the element recognition to detect heavy metal elements/ions and is based on the principles as follows. The first is the formation of a stable DNA-duplex due to the selective binding of specific DNA bases with heavy metal ions; for the second, heavy metal ions aid in the breaking DNAzymes (deoxyribozymes), and finally, the guanine-rich probe switches to a stable G-quadruplex structure [99,100]. Similar to the design for heavy metals detection in environmental monitoring, microbial whole-cell biosensors detect concentrations based on the genetic elements that respond to chemical species. Their effectiveness is determined by the regulatory protein types connected with these promoters, as well as the reporter genes for transcriptional pollutants control [101,102]. A reporter gene in living cells (i.e., microorganisms) is employed as a sensor to convert its biological response into detectable physicochemical signals.

The whole-cell biosensors illustrate a prospective technique for the detection/monitoring of heavy metals and are critical to selectivity and sensitivity [70,100,103]. Using biosensors for continuous and/or individual detection and measurements normally depends on the biologically active element types. Also, biosensors (i.e., organisms) can be integrated into pollutants exposure and used as a method for predicting chemical pollution in the environment. The literature review demonstrated that Shen et al. [104] designed a toxicity MFC system for detecting Cu concentrations of 5–7 mg L^–1^ in a quick speedy response, and Wu et al. [105] also found at a level of 12 mg L^–1^ in their study. Recently, by using the decrease in the cell voltage, the MFC biosensor could monitor arsenic (0.5–5.0 mg L^–1^) and copper (1.0–10 mg L^–1^) with added Cu/As to the anolyte solution [83]. Wu et al. [86] inoculated a facultative anaerobe bacterium into an MFC to investigate an in-situ Cr^6+^ detection sensor. In their research, Wu et al. [105] developed a new sediment MFC-based toxic sensor for online and *in-situ* Cu^2+^ monitoring by using bacteria species such as *Clostridium* and *Geobacter*. A similar study was conducted using *Pseudomonas* and *Geobacter* for in-situ Cr^6+^ detection in real-time from industrial wastewater [106]. Researchers have recently become interested in electro-active biofilms because of their potential applications in environmental monitoring as amperometric biosensors, i.e., in-situ toxicant detection. For the assessment of heavy metal contamination in tap water, an O_2_ reducing microbial cathode-based MFC biosensor was designed, with detection limits in the 1.0 to 10 mg L^–1^ range for ions, including Cr^6+^, Hg^2+^ and Pb^2+^ [107]. As a result, biosensors can accurately reflect the harmful effects of numerous contaminants in the environment. This understanding provides strong technical solutions for online, direct pollutant detection (i.e., heavy metals) and the establishment of an early warning sensor system. Table 2 summarizes the materials and performance of biosensors for heavy metals monitoring.

**Table 2.** Biosensors for heavy metal monitoring with detection limits and response time.

Heavy metals	Configuration of biosensors	Electrodes/Materials		Environmentalmatrix	Detection limits	Response times	Remarks	Refs.
		Anode	Cathode					
Cu, As, Cr, Hg	Double chamber MFC	Carbon fiber	Carbon fiber	Municipal wastewater	0.5–10 mg L^–1^	5 min	MFC as a heavy metal biosensor	[83]
Pb, etc.	A dual-chamber self-powered MFC	Graphite felts	Graphite felts	Water containing heavy metal ions	2 mg L^–1^	N/A	A simple, cheap and sensitive	[101]
Cr	Sediment MFC	Carbon cloth	Graphite felt	Industrial wastewater	0.2–0.7 mg L^–1^	18.3 min	Real-time in-situ detection	[106]
Hg	Doublechamber MFC	Graphitefelt	Graphitepaper	Artificial water	200 mg L^–1^	N/A	Environmentally friendly method	[108]
Pb, Cd, Cu	Magnetoelastic (ME) biosensor	ME ribbon		Heavy metal ions solutions	0.23–0.33 µM	5 min	High-sensitive	[109]
Pb	Micromechanicalbiosensor	A silicon membrane		Lead ions solutions	10 µM	N/A	Based on 8–17 DNAzyme	[130]
Hg, Ag, Cu, Pb	DNA-based electrochemical biosensors	Gold, SPGE, PGE, ITO, magnetic beads, etc.		Environmental matrices	0.0001–300 nM	N/A	Providing a platform for heavy metal detection	[99]
Cu, Cr, Zn, Ni	Reusable single-chambered cylindrical MFC	Graphite sheet	Graphite sheet	Industrial wastewater	5–20 mg L^–1^	N/A	Cost-effective	[100]
Cd, Cu,Zn	Whole-cell bacterial biosensor	Sol-gel matrices (a thin film of sol-gel)		Soilenvironment	1.42 × 10^−4^–3.16 × 10^−4^ mg L^–1^	4–7 h	A green fluorescent protein	[40]
Hg	DNA-based electrodes	Gold nanoparticles, MWCNT		Water samples	1 × 10^−6^ nM	N/A	A time and cost-effective	[127]
Se, Sn, Cr	Bacterial molecular biosensors	*Acinetobacter baylyi ADP1 Tox2*		River water	Low	30 min	Respond at low levels	[71]
Hg, Zn, Cu, Cd	A bacterial whole-cell biosensor	*A. baylyi Tox2*biosensor		Seawater	0.02–1.0 mg L^–1^	A quick	Rapid detection, sensitive biosensor	[70]
Cu, Hg, Zn, Fe, Cr, Cd	MFC-based biosensors	Graphite, carbon cloth, stainless steel	Graphite, carbon cloth, platinum	Water, wastewater, sewage sludge	0.0125–200 mg L^–1^	Short time	Cost-effective	[110]
Cu	A single-chamber MFC	Carbon brush	Carbon cloth	Artificial solution	0.5–12.5 mg L^–1^	N/A	A cost-effective	[105]
Pb	Light-up biosensor	Micro-spin column (Bio-Rad)		Water, serum samples	60.7 nM	N/A	A simple, facile, and cost-effective	[111]
Hg	SERS-based biosensor	Magnetic nanoparticles, SWCNT substrate and CoFe_3_O_4_@Ag		Aqueous solution	8.4 × 10^−4^ nM	N/A	Good sensitivity, selectivity, simplicity, and rapidity	[112]
Pb, Ni, Cd, etc.	Biosensor cells	A novel marine luminescentbacterium *Vibrio sp. MM1*		Aquatic environments	0.97–14.54 mg L^–1^	N/A	High sensitivity	[78]
Hg	DNAzyme-based biosensors	Nanomaterial-assisted DNAzymes		Tap, river, lake, wastewater, etc.	5 × 10^−5^–100 nM	N/A	Highly selective and sensitive recognition	[103]
Pb	A self-cleaning electrochemical biosensor	Cu-TCPP nanofilm and DNAzymes		Artificial solution	1.7 nM	N/A	High sensitive and selective	[123]
Hg, Ag	Novel conductometric biosensor	Three-enzyme system (invertase, mutarotase, glucose oxidase)		Artificial solution	1.25 mM	10–20 min	High sensitivity	[113]
Cd	A novel signal amplification biosensor	Agarose gel electrophoresis, oligonucleotides DNA		Artificial solution	19.93 nM	N/A	A satisfactory recovery rate, high sensitive	[138]
Hg	Whole-cell biosensors	A gas reporting biosensor system		Artificially contaminated soil	5 μM	45 min	On-site detection, saving time and costs	[102]

Remarks: MFC: Microbial fuel cell, PGE: Polycrystalline gold electrode, SPGE: Screen-printed gold electrode, ITO: Indium tin oxide, ME: Magnetoelastic, DNA: Deoxyribonucleic acid, MWCNT: Multi-walled carbon nanotube, SERS: Surface enhancement Raman spectrum, SWCNTs: single-walled carbon nanotubes, TCPP: Tetrakis (4-carboxyphenyl) porphyrin.

Several biosensors based on electrochemical, colorimetric, and fluorescence measurements were successfully devised for heavy metal detection [114–116]. It has been suggested that the nanomaterials such as metal oxide and nanostructured carbon could be effectively used in biosensors development [5,117–119]. In addition, several nanomaterials with heterostructure such as α-Fe_2_O_3_–g–C_3_N_4_, V_2_O_5_ g–C_3_N_4_, and CuS–WuO_3_ were developed, indicating the potential use in electrochemical biosensors [120–122]. For electrochemical sensors, nano-scale structures are attractive materials [123], and their huge specific surface can enhance enzyme, signaling molecule, and catalyst immobilization. Applying electrochemical DNA biosensors for heavy metals detection includes implementing core-shell nanoparticles, nicking enzyme–assisted amplification, and nanocomposites modification [124,125]. Apart from these, applications of nanomaterials based on electrochemical nucleic acid (NA) biosensors for quantitative and qualitative analysis/monitoring of environmental pollutants, e.g., heavy metals have been discussed [126,127]. Table 2 demonstrates how biosensors were classified depending on the recognition component that was used for detecting heavy metals. For example, the bioavailable copper ions in synthetic samples were monitored by biosensor used as an optical transducer and immobilized engineered bacterium *Alcaligenes eutrophus* (AE1239), with the limit of detection (LOD) being 1 μM [128]. DNA-based electrodes biosensor is an excellent candidate for Hg detection, with a LOD of 3 fM [127]. Another DNA biosensor using electrode modification with Fe_3_O_4_@3D-GO reached an excellent LOD for Ag^+^ ion detection, which was equal to 2.0 pM and in the wide range of 0.01–100 nM [129]. A self-cleaning electrochemical biosensor based on DNA nanomotors, novel super G-quadruplex (G4), and 2D Cu-porphyrin (Cu-TCPP) metal-organic nanofilms was designed for the cyclic detection of Pb^2+^ ions, which the LOD was equal to 1.7 nM, and the linear range of 5 nM–5 μM [123]. The synergistic effects of G4-hemin DNAzymes and Cu-TCPP, which has efficient and catalytic H_2_O_2_ reduction, resulting in better performance of electrochemical biosensors [123].

DNA biosensors focusing on the recent design of selective and sensitive detection of heavy metal elements/ions by electrochemical transduction are depicted in Table 2. DNA biosensors used electrochemical transduction to provide a suitable platform for early monitoring/detection of heavy metal ions. Many effective DNA biosensors based on various electrochemical redox indicators have been applied for Ag^+^, Hg^2+^, Pb^2+^ ions detection [99]. A bacterial whole-cell biosensor (*Acinetobacter baylyi* Tox2) was designed to detect heavy metal cytotoxicity in the polluted seawater sources [70]. Consequently, A. *baylyi* Tox2 exhibits excellent application as a sensitive and quick biosensor for investigating cytotoxicity in the marine environment. Many detection methods have been assessed using the 8–17 DNAzyme specific for lead ions [115,130,131]. Shen et al. [132] used DNA-Au bio-barcode based on a signal amplification assay for Pb^2+^ ion detection. Thus, biosensors have attracted much interest for biomonitoring purposes, especially of heavy metal ions.

Results found that Hg^2+^ concentrations analyzed by biosensor could be confirmed by standard methods (e.g., Inductively coupled plasma mass spectrometry, ICP-MS), showing that the biosensor was reliable and relevant for Hg^2+^ ion detection in real samples [133]. For instance, autocatalytic DNA circuit based on exonuclease III and G-quadruplex DNAzyme for mercury ions detection achieved high selectivity and sensitivity. For an LOD, it was 10 fM and the linear range was from 10 fM to 100 nM [134]. Shi et al. [133] used a biosensing system for detecting Hg^2+^ in tap and lake water samples with a 3D graphene/gold electrode and a reporter probe attached to Au nanoparticles. An excellent widely linear range from 0.1 fM to 0.1 µM was reached. In contrast, Hg^2+^, Pb^2+^ and Cd^2+^ levels are strictly monitored by the European Union [103] due to these substances’ highly toxic, bioaccumulative properties, and their impact on human health and the environment [133]. According to the U.S. Environmental Protection Agency (EPA) regulations, the maximum levels of heavy metals such as Hg^2+^, Pb^2+,^ and Cd^2+^ in drinking water are 10 nM, 72 nM and 45 nM, respectively, so these described biosensors could effectively monitor their concentrations [103,116]. The LOD of these heavy metals are detected by biosensors lower than the toxicity safety levelfor example, in drinking water to monitor the quality. Shown here is the critical role of advanced biosensors for heavy metals detection/monitoring to meet requirements, such as monitoring Hg^2+^ concentration in drinking water (i.e., level of 10 nM), following the U.S. EPA standard [134].

Several studies have used eukaryotic microorganisms to investigate whole-cell biosensors for environmental pollution monitoring of heavy metals in aquatic habitats or soils [135]. Yeasts, ciliated protozoa, and microalgae are generally selected as the three main eukaryotic taxonomic groups. For example, a new conductometric biosensor based on alkaline phosphatase activity was developed utilizing immobilized whole-cell microalgae to detect cadmium ions in aquatic environments and habitats [136]. A novel whole-cell microbial biosensor was invented for rapid on-site detection related to Hg contaminated soil with gas signals [102]. This technique could detect bioavailable mercury levels within 45 min and a range from 5 to 500 μM, effectively showing how much pollution there was in the soil. Guo et al. [137] illustrated that a fluorescent whole-cell biosensor could detect mercury (Hg^2+^) contamination in cosmetics with the detection range from 50 nM to 10 μM with incubating time for two hours. It means that biosensors can be used for many purposes and in many industries.

Biosensors could detect and provide information quickly about the toxic pollutants and contamination zone, which is necessary for good environmental management and monitoring. Another advantage of biosensors over traditional analytic techniques is related to their mobility, making possible the measurement of in-situ pollutant levels without added chemical agents and sample preparation. Biosensors can detect and conduct single measurements or continuous real-time monitoring during analysis processes. DNA biosensors based on electrochemical transduction, constitute an sensitive technique and affordable method for detection and monitoring of heavy metal elements [99,138]. Biological sensors are promising in heavy metal ions detection, especially DNA. DNA and DNAzymes are biodegradable compositions, exhibit high selectivity, the advantage of a portable analytical device and in-vitro technique, as well. Based on the highly selectivity of DNAzymes (as a biological recognition element), electrochemical biosensors can determine heavy metal compounds. DNA is easy to synthesize, low-cost, stable at room or higher temperatures, and is an ideal material for biosensors [134]. It shows novel advantages with excellent potential in toxicity determination, i.e., sensitive, cost-effective devices, rapid response to the toxin and short life cycle [71,139,140].

The excellent sensitivity of DNA-based or DNAzyme-based biosensors on heavy metals was indicated by its low LOD (below nM), especially those integrating nanomaterials (Table 2). However, the cell-free biosensor could not provide the information of bioavailable metals in the environment. In addition, the high cost and requirements of advanced technology of nanomaterials might impede the production and application of these cell-free biosensors in developing countries. On the other hand, although the whole-cell or MFC-based biosensors could reveal the bioavailable heavy metals in the samples with lower cost, the sensitivity of them are mostly lower than DNA-based biosensors, limiting their commercial feasibility.

### 4.2 Organic pollutants monitoring

Anthropogenic activities ensure that the natural environment is contaminated by organic pollutants. A wide range of contaminants originates from different industrial, household and agricultural activities. Agricultural waste organic herbicides and pesticides contain toxic compounds mostly found in wastewater, and the compounds are widely used to remove weeds, pests and unwanted vegetation. Industrial wastewater contains organic matter carrying various hydrocarbons, chlorine compounds, aromatic substances and surfactants. Persistent organic pollutants (POPs) including polychlorinated biphenyls (PCBs), polybrominated diphenyl ethers (PBDEs), polybrominated biphenyls (PBBs), phthalate esters (PAEs), and other hazardous pollutants exist in industrial wastewater [141–143]. Household wastes also contribute to organic pollutants in wastewater such as phenyl ether which is found in everyday household products such as soaps, deodorants, plastics and cosmetics [144]. Monitoring the particular organic matter in wastewater is an important aspect of human health and the environment, particularly wastewater treatment and water reclamation processes. The application of biosensor for detecting organic contaminants in wastewater achieves the fastest and most accurate results compared to other traditional methods [145]. Biosensor application can be used for environmental organic pollutants including endocrine disrupting chemicals (EDCs), PCBs, POPs, total petroleum hydrocarbons (TPHs), polycyclic aromatic hydrocarbons (PAHs) and volatile organic compounds (VOCs) (see Table 3).

**Table 3.** Summary of organic pollutant biosensors with detection limits.

Pollutants	Classification	Configuration of biosensors		Detection limit	Remarks	Refs.
		Sensing element	Signaling element			
Bisphenol A	EDC	Enzyme (tyrosinase)	Amperometry/MWCNT modified	0.01 nM	Promising reliable	[146]
Bisphenol A	EDC	Enzyme biosensor	Tyr – AuNPs/Graphite oxide	1 nM	Excellent performanceLong-term stabilityPromising on-site analysis	[147]
Bisphenol A	EDC	Labeled antibody	Fluorimetry	0.014 μg L^−1^	Satisfactory repeatabilityGood agreement	[148]
Bisphenol A	EDC	Sol–gel films	Tyr/TiO_2_-MWCNTs-PDDA- Nafion/graphite	66 nM	High sensitivity Good precision	[149]
Estrogen Estradiol Phenol Nonylphenol Bisphenol A	EDCs	Enzyme (tyrosinase)	Amperometry	4.4 mg L^−1^4.06 mg L^−1^0.94 mg L^−1^2.2 mg L^−1^5.2 mg L^−1^	FastGood sensitivity	[150]
Estrone	EDC	Labeled antibody	Fluorimetry	0.2 ng L^−1^	Fast, sensitive, and cost-effective detectionA powerful tool in aquatic analytics	[151]
Phenol	EDC	Whole-cell (*Acinetobacter* sp. DF4)	Light emission/bioluminescence	2.5 mg L^−1^	Response to phenol-contaminated soils	[152]
Triclosan	EDC	Molecular imprinting polymers (MIP)	Surface plasmon resonance (SPR)	0.017 μg L^−1^	Excellent performanceFast, sensitive and simple nanosensor	[144]
AtrazineBisphenol AEstroneIsoproturon	HerbicideEDCEDCHerbicide	Labeled antibodies	Total internal reflection fluorescence (TIRF)	0.002 μg L^−1^0.005 μg L^−1^0.019 μg L^−1^0.016 μg L^−1^	Low-costEarly warning application	[153]
Propanil	Herbicide	Polyclonal IgG antibody	Fluorimetry	0.6 ng L^−1^	Fast, sensitive, and cost-effective detection	[154]
Acetamiprid	Pesticide	Electrochemical DNA Aptasensor	Ferrocene (Fc)- based hollow polymeric nanospheres (Fc-HPNs)	3.3 × 10^−7^ nM	Excellent sensitivity	[155]
Carbaryl	Pesticide	Monoclonal antibody	Surface plasmon resonance (SPR)	1.38 μg L^−1^	Useful and portable for on-line monitoring	[156]
Organophosphorus (OPs)	Pesticide	A portable LIG-based electrochemical biosensor	MnO_2_-bridged enzyme-aided signal amplification	1.2 μg L^−1^	Good performance for pesticideApplications in the environment and food safety fields	[157]
Pyrethroid	Pesticide	Whole-cell and antibody	Calorimetry	3 μg L^−1^	Simple and portable for on-site colorimetric applicationFunctional at least 90 days after lyophilization	[169]
NaphthalenePhenanthrene	PAHs	DNA/Sol–gel array	Fluorimetry	0–10 mg L^−1^	Effective PAHs detection in water, and biological samples	[158]
Polybrominated diphenyl ethers (PBDEs)	POP	Whole-cell (*Sphingobium xenophagum*)	Bioluminescence	10 nM	Novel PBDE-specific sensing element	[91]
PCB-28PCB-101	PCB	Enzyme (peroxidase)/immobilized polyaniline modified Pt electrode	Amperometry	0.016 μg L^−1^0.019 μg L^−1^	Useful in POPs detection in landfill leachates	[159]
TPH	TPH	Antibody and enzyme	Immunoassay	25 mg kg^−1^	Quick on-site useDetect TPH in soil and water	[160]

Remarks: TIRF: Total internal reflection fluorescence, SPR: Surface plasmon resonance, OPs: Organophosphorus, LIG: Laser-induced graphene, TIRF: Total internal reflection fluorescence, MIP: Molecular imprinting polymers, MWCNT: Multi-walled carbon nanotube, EDC: Endocrine disrupting chemical, PCBs: Polychlorinated biphenyls, POPs: Persistent organic pollutants, PAHs: Polycyclic aromatic hydrocarbons, TPH: Total petroleum hydrocarbons.

Advances in the field of biotechnology have enabled biological materials to function as receptors, so these bioreceptors can analyze VOCs with high selectivity and sensitivity [161]. Some applications can detect VOCs based on protein and peptides biosensors, such as sensitive olfactory biosensors having a remarkable performance for high selectivity and sensitivity at very low concentrations by using odorant-binding protein (OBP) operated as a sensing component [162]. In addition, the nonspecific biosensor based on microbial electrochemical cell-based (MXC) structure can be successfully used to monitor PCBs and PAHs such as toluene [163]. For specific targeting of benzene, phenol, toluene, and other related pollutants, engineered whole-cell biosensors based on the metabolic genes as sensing elements were developed [164,165]. Other substances including pesticides and EDCs could be detected in river water and wastewater by biosensors especially nanomaterials-based ones, suggesting their feasibility in real environmental samples [166].

Currently, the successful application on VOCs and PAHs such as alkanes, toluene, naphthalene in wastewater, seawater, and soil using whole-cell luminescence biosensor has been reported [8]. However, limited information about the key sensing element based on the metabolic mechanisms of microorganisms constrain the development of biosensors for specific organic pollutants such as emerging contaminants [91]. Recent research has highlighted the importance of high-throughput sequencing and synthetic biology tools on biosensors for organic pollutants since these techniques provide more genetic information about sensing elements [91,167]. Additionally, the usage of two different fluorescence as signaling elements enables the biosensor to detect different types of hydrocarbon simultaneously [165]. For simple and rapid on-site use, the colorimetric biosensors for pyrethroid insecticide and halogenated hydrocarbons performed well in testing samples [168,169]. So it can be states that a biosensor for detecting organic pollutants in the environment is now more efficient and feasible in rapid on-site screening. It complements traditional chemical analysis for large-scale monitoring.

Nowadays widespread industrial spillage is the major problem causing environmental degradation or destruction. For unique applications, biosensors can be personalized such as TPHs screening kit which is a favored tool for monitoring analytes on site in different media (soil, water and vapor) [170]. The immunoassay-based TPHs kit founded on antigen-antibody and enzyme is a biosensor due to its biological sensing elements. It has been suggested that TPHs immunoassay is one of the most widely used methods to quickly detect petroleum compounds especially in soil samples [171,172]. The low-cost and quick on-site utility are the main strengths of using the TPHs immunoassay kit [172]. However, the drawbacks of interference of media and nonspecific target were reported in TPHs assay kit, which is similar to other biosensors [171,172]. For this reason, the TPHs immunoassay could be used as large-scale screening method prior to precise quantification of TPHs using instruments such as GC. It could reduce the amount of energy consumed and carbon footprint as required by the SDGs.

An overview of the current achievements of biosensors and those devised to monitor organic pollutants was done in this section. Monitoring of organic contaminants plays an important role in the protection of human health and the ecosystem. By applying a biosensor, the rapid detection and screening of organic contaminants can be achieved. These biosensors are quite inexpensive, portable, available for on-site use, and no waste is generated during analysis. However, there are some limitations of these biosensors such as sensitivity and selectivity of the target pollutants, limiting applicability. Some challenges need to be overcome for biosensors including interference of humidity, effect of pH on sensitivity and selectivity, enhancing the sustainability of sensing components of biosensors, and ensuring the adaptation of nanomaterials, which can improve the performance of biosensors. More knowledge of metabolic genes for POPs is needed for further improvement of sensing elements. Thus, current biosensors technology could be utilized in monitoring EDCs, PCBs, POPs, TPHs, PAHs, and VOCs, while those difficult-to-degrade organic pollutants might not be efficiently monitored.

Similar to the biosensor detecting heavy metals, the cell-free biosensors using enzyme, DNA, and aptamer showed high sensitivity to organic pollutants including EDCs, pesticides, and PCBs (Table 3). However, the lack of bioavailability data and higher requirements of nanomaterials are also the limitations of cell-free biosensors. The whole-cell biosensors detecting organic pollutants are relatively few, which is due to the low biodegradability of the organic pollutants that hinders their development. In addition, most of the whole-cell biosensors have higher LOD than cell-free biosensors, showing the lower sensitivity (Table 3). Currently, the commercial whole-cell biosensors are mainly nonspecific and toxicity-based with the bioluminescence signals, whereas the biosensors specific to heavy metals and organic pollutants (TPHs) are all cell-free biosensors with DNAzyme, enzyme, and antibody (Table 4).

**Table 4.** Commercial biosensors and the product details.

Product name	Analyte	Sensing element	Signaling element	Manufacturer (country)
Microtox	Toxicity	Whole-cell	Bioluminescence	Azur Environmental (US)
LUMIStox	Toxicity	Whole-cell	Bioluminescence	Hach (US)
ToxAlert	Toxicity	Whole-cell	Bioluminescence	Merck (Germany)
Catalytic DNA Sensors	Metals	DNAzyme	Fluorescence	Quasar Instruments (US)
PETRO RISc Soil Test	TPHs	Enzyme and antibody	Color	Ensys Energy Systems (US)
EnvironGard Petroleum Fuels in Soil	TPHs	Enzyme and antibody	Color	Merck Millipore (Germany)

In short, establishing monitoring activities based on biosensors can be applied for the significant recognition/detection of heavy metals, organic chemicals, microorganisms, etc. Biosensors are robust environmental monitoring solutions with important novel characteristics and advantages [173]. Biosensors are early detection techniques with much promise in achieving on-site monitoring and continuous real-time environmental data. Wastewater and water monitoring biosensors are an innovative approach for the ultimate goal of on-site monitoring within the framework of the UN’s Sustainable Development Goals (SDGs).

### 4.3 Wastewater quality monitoring and environmental viability

Wastewater discharge is the one of the source of environmental pollution, and the wastewater quality monitoring could be also achieved by the biosensors [145]. The biosensors especially MFC-based were applied in wastewater monitoring globally. In Asia Pacific countries, it has been reported that Au^3+^ ions in wastewater from Taiwan could be detected by engineered bacteria [174]. In addition, The MFC-based BOD biosensor was used in monitoring swine wastewater in Japan, which minimizes the energy cost for aeration [175]. The multiple heavy metals and phenols in real wastewater samples from China were able to be detected by MFC-based biosensors [176]. The immobilized engineered bacteria were used in screening PAHs in industrial wastewater in India [177]. The toxic metals (Cr^6+^ and Fe^3+^), NO_3_^−^, and sodium acetate in the wastewater from Connecticut (USA) could be assessed by the batch mode MFC-based biosensors [178]. Similarly, the brewery wastewater obtained from Canada containing COD and others such as NH_4_^+^ was shown to successfully monitored by MFC-based biosensors [179]. The MFC-based biosensors were also used in measuring the BOD in rice washed wastewater in Ecuador [180]. In South Africa, it has been shown that the influents and effluents from wastewater treatment plants were assessed by nonspecific whole-cell bioluminescence biosensors [181]. In Europe countries, the MFC-based biosensors were applied in detecting the BOD in domestic and brewery wastewaters in Hungary [182]. The COD in domestic wastewater from Spain discharged into constructed wetlands could be monitored by MFC-based biosensors [183]. Taken together, the MFC-based biosensors are broadly applied in wastewater quality monitoring due to its strengths of saving energy and continuous monitoring, which could correlated to the SDGs 6 (clean water and sanitation). However, further studies on detecting specific pollutants such as toxic metals in industrial wastewater are required for better wastewater quality. The performance and application of biosensors in real environmental samples such as river, wastewater, and soil samples were suggested in recent studies (Tables 2 and 3). For better commercial and environmental feasibility, more research about the real environmental sample tests is needed.

## 5. Biosensor for Sustainable Development Goals (SDGs)

Biosensors can detect impurities in the air, soil or water and in this way lead to finding the main sources of pollutants by implementing activities that mitigate impurities and help realize the SDGs, especially SDG 6 (clean water and sanitation), 12 (responsible consumption and production), 13 (climate action), 14 (life below water), and 15 (life on land). In developing countries, the environmental pollution might raise the health concern such as As contamination in groundwater, drinking water, and irrigation water, highlighting the needs for screening and monitoring the contaminants [184]. Compared with traditional environmental analysis methods, biosensors for environmental monitoring possess characteristics such as low-cost, minimal technical expertise and sample pretreatment, and feasibility for on-site use, saving energy and nonuse of hazardous materials [167]. For instance, the color-based arsenic biosensor on paper strip can be easily used in drinking water by naked eyes without complicate instruments [14]. Therefore, applying the biosensors with such advantages on environmental monitoring could help achieving SDG 6 (clean water and sanitation) by accomplishing large screening of pollutants for clean water and improving the access to affordable drinking water especially in developing countries (Table 5). Furthermore, the SDG 6 (clean water and sanitation) and its target 6.3 (water quality and wastewater) could be achieved by using biosensors in simultaneous monitoring of multiple toxic metals in polluted rivers before human health is affected [71]. Water quality monitoring such as BOD, COD, and toxicants using the MFC-based biosensors and the toxicity-based nonspecific biosensors reflects not only the toxicity in drinking water but also the extent of eutrophication [23,81]. Targeted here are both SDGs 6 and 14 (life below water) which refers to detecting the potential pollution in freshwater and marine ecosystems as well as drinking water. For terrestrial ecosystems this refers to SDG 15 (life on land), and the soil contaminants-detecting biosensor could be applied to screen potential soil pollution, which can guide what happens in terrestrial ecosystems [185,186].

**Table 5.** The relationship of SDGs and biosensor application.

SDGs	Contributions of biosensor application
SDG 6: clean water and sanitation	Large screening of environmental pollutants in waters for reducing the pollution in drinking water and aquatic ecosystemsImproving the access to safe and affordable drinking water due to ease of use and portability
SDG 12: responsible consumption and production	Reducing the hazardous chemicals and wastes in sample pretreatment and instrumental analysisBetter management of production and consumption in environmental monitoring to prevent the adverse impacts on human health and the environment
SDG 13: climate action	Reducing the energy and corresponding carbon footprint from traditional chemical analysis
SDG 14: life below water	Help managing the pollution into marine ecosystems
SDG 15: life on land	Help managing the pollution into terrestrial ecosystems

Furthermore, the results of biosensors provide more information about bioavailability and toxicity, which can complement traditional chemical analysis methods. From the sustainability point of view, biosensors’ deployment reduces the amount of hazardous chemicals that are used in operational and sample pretreatment processes, which is also related to SDG targets 6.3 and 12.4 (reducing the hazardous waste produced) [167]. Portability and on-site monitoring feasibility using the biosensors reduce the carbon footprint which is a consequence of transportation. In addition, the large-scale screening of biosensor environmental analysis helps with precise measurements and saves electricity and energy [167]. It has been reported that chemicals and energy used in laboratories did make a substantial contribution to the carbon footprint and green GHGs [20–22]. A summary of the relationship between SDGs and biosensor application is shown in Table 5. The complementary use of biosensors and traditional chemical analysis methods could realize SDG 13 (climate action) by reducing energy consumption and the carbon footprint.

## 6. Challenges of biosensor application on SDGs achievement

To provide reliable environmental monitoring results with low carbon footprint and less hazardous waste for achieving SDGs, biosensors need to be more feasible for quick-screening and on-site usage. The progress of biosensors which involves better selectivity, limits of detection and sensitivity is improving in the way that contaminants in the environments are detected, promoting a clean and green environment, while the challenges of sustainability, portability, and reusability still remain. Researchers want to resolve these challenges to enhance the biosensor application at advanced levels [187]. Currently, several advanced nanomaterials have been applied in the electrodes of cell-free and whole-cell biosensor to improve pollutants detection [188]. For cell-free biosensor, aptamer coupled with nanomaterials biosensors were commonly used for their good selectivity and reliability [29]. However, the cost for nanomaterials and aptamer-based biosensor production means they are still not commercially feasible, limiting their environmental application. The energy and carbon footprint from manufacturing these biosensors with nanomaterials and high performance might compromise their contribution to the SDGs. The possible risk of environmental nanomaterial use is still unclear so further information about the safety of nanomaterial-based biosensor and lowering the costs and energy consumption is needed. Recent studies mostly focused on sensing and signaling elements optimization, whereas the practicality tests using real wastewater samples are still rarely undertaken. More studies using biosensors in real environmental samples to assess the influence of other interfering substances such as organic matter chelation are warranted.

## 7. Conclusions and future prospects

Recognizing the growing call for more environmentally, economically, and socially responsible societies, emerging remediation technologies and governing strategies are being developed in alignment to the sustainable future. Biosensors using biological sensing elements coupled with signaling elements can be used to detect impurities in environmental samples so that the responsible sources of pollutants are known. By implementing remedial activities to remove harmful impurities from the sources, the UNSDG goals can be achieved. Despite the potential contribution to SDG achievements, few research reported the importance of biosensor application on SDGs, and the correlation of environmental monitoring using biosensors and SDGs. Herein, this article reviewed the current progress being made in biosensors for the purposes of environmental monitoring and their contribution to achieving the UNSDGs, including clean water and sanitation (SDG 6), responsible consumption and production (SDG 12), climate action (SDG 13), saving life below water (SDG 14), and saving life on land (SDG 15). While bio-sensing technologies have advanced significantly in the past decades, further research on how to improve the sensitivity, stability, safety, and portability is warranted for environmental monitoring biosensors and their future applications.

Cell-free biosensors are powerful in terms of their quick and specific response to environmental pollutants, whereas a whole-cell biosensor can provide additional information about the bioavailability and toxicity that cannot be analyzed by cell-free biosensors. The results from nonspecific whole-cell biosensors directly reflect the total impact of pollutants on the test microorganisms, which is mostly based on the inhibition of microbial functions. However, nonspecificity is the main weakness when designing a specific targeting policy. On the other hand, the specific whole-cell biosensor can provide good information about the target pollutants, yet the sensitivity and stability still need to be improved for feasible commercial application. Recently, genetic methods have been developed in whole-cell biosensor to improve its sensing and signaling elements for better sensitivity. However, research into environmental sample tests, storage time such as shelf life, and safety of genetically modified biosensors is needed.
